# Exploring the nature of prediagnostic blood transcriptome markers of chronic lymphocytic leukemia by assessing their overlap with the transcriptome at the clinical stage

**DOI:** 10.1186/s12864-017-3627-4

**Published:** 2017-03-20

**Authors:** Jelle Vlaanderen, Max Leenders, Marc Chadeau-Hyam, Lützen Portengen, Soterios A. Kyrtopoulos, Ingvar A. Bergdahl, Ann-Sofie Johansson, Dennie D.G.A.J. Hebels, Theo M.C.M. de Kok, Paolo Vineis, Roel C.H. Vermeulen

**Affiliations:** 1 Institute for Risk Assessment Sciences (IRAS), Utrecht University, Utrecht, The Netherlands; 2 Department of Epidemiology and Biostatistics, School of Public Health, Imperial College London, London, UK; 3 Institute of Biology, Medicinal Chemistry and Biotechnology, National Hellenic Research Foundation, Athens, Greece; 4 Department of Biobank Research, University of Umeå, Umeå, Sweden; 5 Department of Oncology, University of Umeå, Umeå, Sweden; 6 Department of Toxicogenomics, Maastricht University, Maastricht, The Netherlands; 7 Molecular and Genetic Epidemiology, Human Genetics Foundation (HuGeF), Turin, Italy; 8 Julius Center for Health Sciences and Primary Care, University Medical Center Utrecht, Utrecht, The Netherlands

**Keywords:** B-cell lymphoma, Chronic lymphocytic leukemia, Transcriptomics, Prediagnostic study, Public data

## Abstract

**Background:**

We recently identified 700 genes whose expression levels were predictive of chronic lymphocytic leukemia (CLL) in a genome-wide gene expression analysis of prediagnostic blood from future cases and matched controls. We hypothesized that a large fraction of these markers were likely related to early disease manifestations. Here we aim to gain a better understanding of the natural history of the identified markers by comparing results from our prediagnostic analysis, the only prediagnostic analysis to date, to results obtained from a meta-analysis of a series of publically available transcriptomics profiles obtained in incident CLL cases and controls.

**Results:**

We observed considerable overlap between the results from our prediagnostic study and the clinical CLL signals (*p*-value for overlap Bonferroni significant markers 0.01; *p*-value for overlap nominal significant markers < 2.20e-16). We observed similar patterns with time to diagnosis and similar functional annotations for the markers that were identified in both settings compared to the markers that were only identified in the prediagnostic study. These results suggest that both gene sets operate in similar pathways.

**Conclusion:**

An overlap exists between expression levels of genes predictive of CLL identified in prediagnostic blood and expression levels of genes associated to CLL at the clinical stage. Our analysis provides insight in a set of genes for which expression levels can be used to follow the time-course of the disease; providing an opportunity to study CLL progression in more detail in future studies.

**Electronic supplementary material:**

The online version of this article (doi:10.1186/s12864-017-3627-4) contains supplementary material, which is available to authorized users.

## Background

Recently, Chadeau-Hyam et al. [1], performed the first large-scale prediagnostic analysis of blood-derived genome-wide gene expression profiles in relation to future risk of B-cell lymphomas from 263 cases and 439 controls. Over 700 genes - mostly involved in B-cell signaling and the regulation of the immune system - were found to be differentially expressed in blood samples of participants who were later in life (between 1 and 17 years; median 6.9 years) diagnosed with chronic lymphocytic leukemia (CLL). Logistic models including 20 genes from the originally 700 identified genes indicated excellent predictive performances with areas under the curve ranging between 89 and 96% [1].

When studying the relation between gene expression and subsequent disease in a prediagnostic setting, observed signals can reflect altered disease risk (i.e., markers of susceptibility/vulnerability), reflect etiological pathways leading to disease (i.e., markers of early biologic effect) or, when the latency period of the disease surpasses the time to diagnosis (from blood sampling to clinical diagnosis), can be a result of the disease itself (i.e., markers of disease) [2]. In the study of Chadeau-Hyam et al., it was suggested that the differential gene expression in CLL was (at least partly) caused by the presence of early disease, because the results for CLL agreed with several studies on differential gene expression in CLL using tumor material. Determining whether signals are markers of susceptibility, markers of early biological effect or markers of disease itself would require additional information, such as prior knowledge of the underlying pathways or, ideally, transcriptomic profiles at multiple time points along pathogenesis. The latter could aid in understanding the underlying etiological and pathophysiological pathways as this could enable observation of relevant temporal transcription regulation [3]. In the absence of such longitudinal biological samples, the elucidation of individual transcriptomic trajectories driving future disease risk cannot be directly addressed. However, owing to the wealth of publically available data from established repositories such as Gene Expression Omnibus (GEO) of the National Center for Biotechnology Information (NCBI) [4] and ArrayExpress of the European Bioinformatics Institute (EBI) [5], many data sets from clinical case–control and, to a lesser extent, prediagnostic studies are now available and commonly used for research that goes beyond the scope of their original context [6].

In the current study, we compare results from our initial blood-based prediagnostic genome-wide gene expression study, to those obtained from transcriptomic profiles from clinical cases (i.e., patients whose samples were collected after diagnosis) of CLL and healthy controls. These transcriptomic profiles arose from several studies that used both blood and tumor samples. In order to combine the information from this heterogeneous data set, we used a meta-analytic framework, which eased the comparison with the results from our prediagnostic study. The identification of markers that are specific to clinical and/or prediagnostic studies has the potential to inform on their prediagnostic nature. For instance, markers that are present in both prediagnostic and clinical samples may indicate early disease biomarkers, while markers exclusively found in prediagnostic samples may either indicate susceptibility to disease or biological imprints of disease progression (e.g., monoclonal B cell lymphocytosis), and conversely, exclusive clinical markers may reflect disease manifestation. Pooling sources of prediagnostic and clinical transcriptomic data therefore has the potential to inform on the natural history of the gene expression trajectories involved in the development and progression of CLL and other diseases.

## Methods

### Meta-analysis of clinical studies

Public repositories GEO and ArrayExpress were searched using the terms ‘chronic lymphocytic leukemia’, ‘CLL’, and a series of B-cell lymphoma related search terms: ‘diffuse large B-cell lymphoma’, ‘DLBCL’, ‘follicular lymphoma’, ‘FL’, ‘multiple myeloma’ or ‘MM’. Expression profiling studies on human samples were scanned for relevancy. Studies were considered eligible for inclusion in the meta-analysis if they quantified genome-wide gene expression levels in biological samples of CLL patients (or human lymphomatic cell lines) and healthy controls. Studies that stimulated sampled cells before quantifying expression levels were excluded, as were studies that selected patients based on specific genetic alterations (e.g., gain or deletion of chromosomes or specific mutations). Nine unique clinical studies (i.e., with prevalent cases) were selected for inclusion in the meta-analysis (Additional file 1: Table S1 and Figure S1) [7–13]. Out of these nine studies, most studies (*N* = 6) included peripheral blood samples; the remaining studies sampled bone marrow or lymph nodes.

The *GEOquery* package for R was used to download raw expression and phenotype data for studies that were included in our analysis [14]. Because various (sometimes unknown) preprocessing methods were performed on the data, expression levels were normalized using a rank-based inverse normal transformation. Because different platforms were used, expression data were shrunk such that only one probe per gene was retained. In case of several probes assaying the same gene, we used the median expression levels across all probes as a summary statistic to optimize comparability across the datasets.

A random effects meta-analysis was performed on the downloaded raw data to identify differentially expressed genes. For this, the *MAMA* (Meta-Analysis of MicroArray) package for R was used, which combined standardized effect sizes per gene over the included studies into overall estimates of the average effect size [15, 16]. A Cochran Q-value was also calculated as a measure of heterogeneity in effect size between the included studies. Only genes that were measured in all clinical studies were included in the meta-analysis (*N* = 11,904). To examine the effect of the varying sampling tissues between the studies on the average effect size we performed a sensitivity analysis in which the meta-analysis only included studies that used peripheral blood samples, which was also used in the prediagnostic study.

### Prediagnostic study

We used participant and gene expression level data from the EnviroGenomarkers project (http://www.envirogenomarkers.net). This project aims to discover biomarkers predictive of increased risks of cancer, using data from the Västerbotten Intervention Project (VIP), which is a part of the Northern Sweden Health & Disease Study (NSHDS), and EPIC-Italy cohorts, both described in detail elsewhere [17, 18]. In short, VIP includes approximately 95,000 individuals from the general population of the Västerbotten county (Sweden). Since 1985, all inhabitants aged 40, 50 and 60 are invited for screening. Included participants provided questionnaire data, anthropometric measurements and blood samples and are followed-up for disease outcomes through regional health registries. The EPIC-Italy study is the Italian contribution to the larger European Prospective Investigation into Cancer and Nutrition (EPIC) study, including over 47,000 participants (aged 35–70 years) from five different areas in the country (Florence, Naples, Ragusa, Turin, Varese). At baseline (1993–1998), biological samples were obtained from participants in addition to anthropometric measurements and questionnaire information on diet and lifestyle. Participants were followed-up for diseases through local registries. In a previous paper we demonstrated that high quality RNA expression profiles could be obtained from these prospective collections if blood samples were cold stored (−80, or -196C) within two hours after collection [19].

In total, 39 participants diagnosed with CLL during follow-up were included and matched to 39 controls on sex, age, center, fasting status and date of blood collection in two analytical phases. In addition, 442 blood samples from the same study population of healthy individuals at the time of blood draw, were added as unmatched controls to maximize statistical. RNA was obtained from peripheral blood mononuclear cells (PBMC). Only samples placed in cold storage within two hours after blood collection were included in this study. Genome-wide gene expression was obtained using an Agilent 4 × 44 K human whole genome microarray platform. In total, samples of 39 future CLL cases and 438 controls were successfully analyzed. For the current study, data was collapsed by gene (29,662 probes representing 15,613 genes), using the median level of expression if multiple probes correspond to the same gene, for comparison with the data sets from public repositories. The per-gene data were analyzed using mixed models as used for the original per-probe analyses. In short, the expression level of a gene was modeled as dependent variable and case status, age, gender, country, experimental phase, BMI, education, physical activity, smoking status and alcohol consumption were included as fixed factors. Random intercepts were included for the dates of RNA isolation, hybridization and dye labeling.

### Comparing clinical with prediagnostic markers

**Fig. 1:**
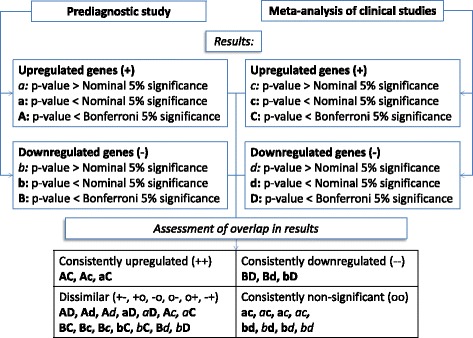
Graphical representation of the approach followed to assess the overlap of gene-expression markers identified in the prediagnostic study with gene-expression markers identified in the clinical study. Based on the overlap between the prediagnostic study and clinical studies, four main categories were defined: consistently upregulated (++), consistently downregulated (−−), dissimilar (+−, +o, −o, o-, o+, −+), or consistently non-significant (oo). The first and second symbols relate to clinical and prediagnostic data respectively, and ‘+’ indicates a positive association, ‘-’ an inverse association, and ‘o’ a non-significant association. A gene was considered differentially expressed in both sections (‘++’ or ‘--’) if it reached Bonferroni-corrected 5% statistical significance in at least one of the two types of studies, and reached nominal 0.05 significance in the other study type. Any gene not reaching Bonferroni 5% statistical significance in either type of study is considered as a null finding (‘oo’)

Based on the overlap between the prediagnostic study and clinical studies, four main categories were defined: consistently upregulated (++), consistently downregulated (−−), dissimilar (+−, +o, −o, o-, o+, −+), or consistently non-significant (oo). The first and second symbols relate to clinical and prediagnostic data respectively, and ‘+’ indicates a positive association, ‘-’ an inverse association, and ‘o’ a non-significant association. A gene was considered differentially expressed in both sections (‘++’ or ‘--’) if it reached Bonferroni-corrected 5% statistical significance in at least one of the two types of studies, and reached nominal 5% statistical significance in the other study type (*p* < 0.05). Any gene not reaching Bonferroni 5% statistical significance in either type of study was considered as a null finding (‘oo’). A graphical representation of this approach is displayed in Fig. 1.

We used Venn diagrams to show the overlap between clinical and prediagnostic markers. The Venn diagrams consist of two concentric semi-circles with the darker shaded outer layer indicating the number of markers that reached Bonferroni significance and the lighter shaded inner layer indicating markers that only reached nominal statistical significance. The red shaded upper semi-circle reflects upregulated markers and the green shaded lower semi-circle reflects downregulated markers.

Further insight was gained by plotting signed *p* values from the prediagnostic study (i.e., significance of case status in the linear mixed model) against those of the meta-analysis (i.e., significance of the combined effect size). The sign of the p-value distinguishes under- from overexpression.

We assessed the probability that the overlap between significant signals from the prediagnostic study and the meta-analysis of clinical studies was due to chance using a Fisher’s exact test (analysis conducted for both the overlap in Bonferroni significant signals and nominal significant signals).

### Functional analyses and bioinformatics

We conducted principal components analysis on the similarly differentially expressed genes (‘++’ and ‘--’) and on the genes exclusively seen prediagnostically (‘o+’ and ‘o-’). We used principle components that explained more than 5% of the total variance in further analyses. We investigated pairwise correlation between principal components to identify potential common latent structures and we examined the association of principal components with time to diagnosis using the linear regression model that was used in the prediagnostic study.

We assessed enrichment of KEGG pathways in our data using gene enrichment analyses on all genes that reached Bonferroni significance in either the prediagnostic or clinical studies. We defined a Bonferroni corrected Fisher’s exact *p* value < 0.05 as cut-off for enrichment. For KEGG pathways that were enriched we compared the proportion of genes included in a pathway across four gene categories defined in our study: similarly differentially expressed genes (‘++’ or ‘--’), genes exclusively seen prediagnostically (‘o+’ or ‘o-’), genes exclusively seen in clinical studies (‘+o’ or ‘-o’) and dissimilarly differentially expressed genes (‘+−’ or ‘−+’), using a Fisher’s exact test. Gene set enrichment analyses was conducted using the *KEGGREST* R package [20].

To gain mechanistic insight into the role of the genes identified in this study in the natural history of CLL, we assessed the occurrence in our results of 44 putative CLL driver genes recently identified through whole-exome sequencing of 538 CLL and matched germline DNA samples [21].

## Results

The meta-analysis of clinical CLL markers included nine studies and a total of 11,904 genes. For 35% of the genes we observed Bonferroni significant heterogeneity in expression between studies, while 116 genes were Bonferroni-significant differentially expressed (See Additional file 1: Table S2 for the top 25 hits from this analysis). When we included only the six studies that used peripheral blood samples in the meta-analysis, we observed between-study heterogeneity in 0.7% of the genes and 6 genes were Bonferroni-significant differentially expressed.

Results of the per-gene replication of the prediagnostic study were very similar to the results of the original per-probe analyses. 535 genes were Bonferroni-significant differentially expressed (See Additional file 1: Table S3 for the top 25 hits from this analysis).

**Fig. 2:**
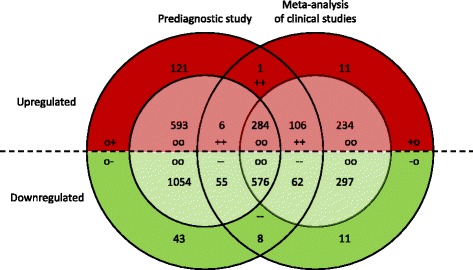
Venn diagram indicating the overlap between genome wide significant genes (*darker shaded*) and nominal significant genes (*lighter shaded*) concurrently upregulated (*red*) or downregulated (*green*) in the prospective and clinical CLL study. Genes with a dissimilar direction of effect between the prospective and clinical CLL study (regardless of the significance level of the association) are not included in this graph. Symbols correspond to the four main categories as defined under Fig. 1

In Fig. 2 we show the overlap between concurrently up- and downregulated clinical and prediagnostic markers. We observed clear overlap in differentially expressed genes. One upregulated gene (COCH) and 8 downregulated genes (ARHGAP32, EFHC2, FAM134B, KLF3, MAFB, RAB33A, SCML1, SMAD7) were Bonferroni-significant differentially expressed in the meta-analysis of clinical studies as well as in the prediagnostic study. Among concurrently upregulated genes, 106 were Bonferroni-significant in the prediagnostic study and reached nominal significance in the meta-analysis of clinical studies, while 6 genes were Bonferroni-significant in the meta-analysis and reached nominal significance in the prediagnostic study. 284 genes reached nominal significance (but not Bonferroni significance) in both the prediagnostic study and the meta-analysis of clinical studies. Among concurrently downregulated genes, 62 were Bonferroni-significant in the prediagnostic study and reached nominal significance in the meta-analysis of clinical studies, while 55 genes were Bonferroni-significant in the meta-analysis of clinical studies and reached nominal significance in the prediagnostic study. Five hundred sevety six genes reached nominal significance (but not Bonferroni significance) in both the prediagnostic study and the meta-analysis of clinical studies. Results from a Fisher’s exact test indicated that the overlap between markers that were significant in the prediagnostic study and markers that were significant in the meta-analysis of clinical studies was unlikely due to chance (*p*-value for overlap Bonferroni significant markers 0.0123; *p*-value for overlap nominal significant markers < 2.2e-16). We provide the full list of concurrently up- or downregulated genes that reached Bonferroni-significance in either the meta-analysis or the prediagnostic study in Additional file 1: Table S4.

**Fig. 3:**
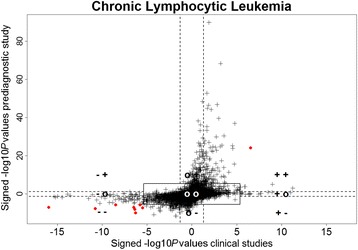
Comparison of differential gene expression observed in the prediagnostic study and the results of the meta-analysis of clinical studies. Solid lines are placed at *p* values of significance threshold after Bonferroni correction. Dashed lines correspond to *p* value = 0.05. Solid red circles represent significant genes after Bonferroni correction in both the prediagnostic study and meta-analysis on clinical studies. Spearman rank correlation between signed –log 10 *p* values was 0.53

Further insight is gained from Fig. 3. We observed a Spearman rank correlation of 0.53 between signed -log10 *p* values from the prediagnostic study and the meta-analysis of clinical studies (*p*-value < 2.2e-16). For 192 genes that were Bonferroni-significant differentially expressed in the prediagnostic study we observed a *p* value above 0.05 in the clinical study (‘o+’ or ‘o-’), and 41 genes vice versa (‘+o’ or ‘-o’). Finally 7 genes that were nominally significantly downregulated in the meta-analysis of clinical studies were nominally significantly upregulated in the prediagnostic study (of which none Bonferroni-significant) and 25 genes that were nominally significantly upregulated in the meta-analysis of clinical studies were nominally significantly downregulated in the prediagnostic study (of which none Bonferroni-significant).

**Fig. 4:**
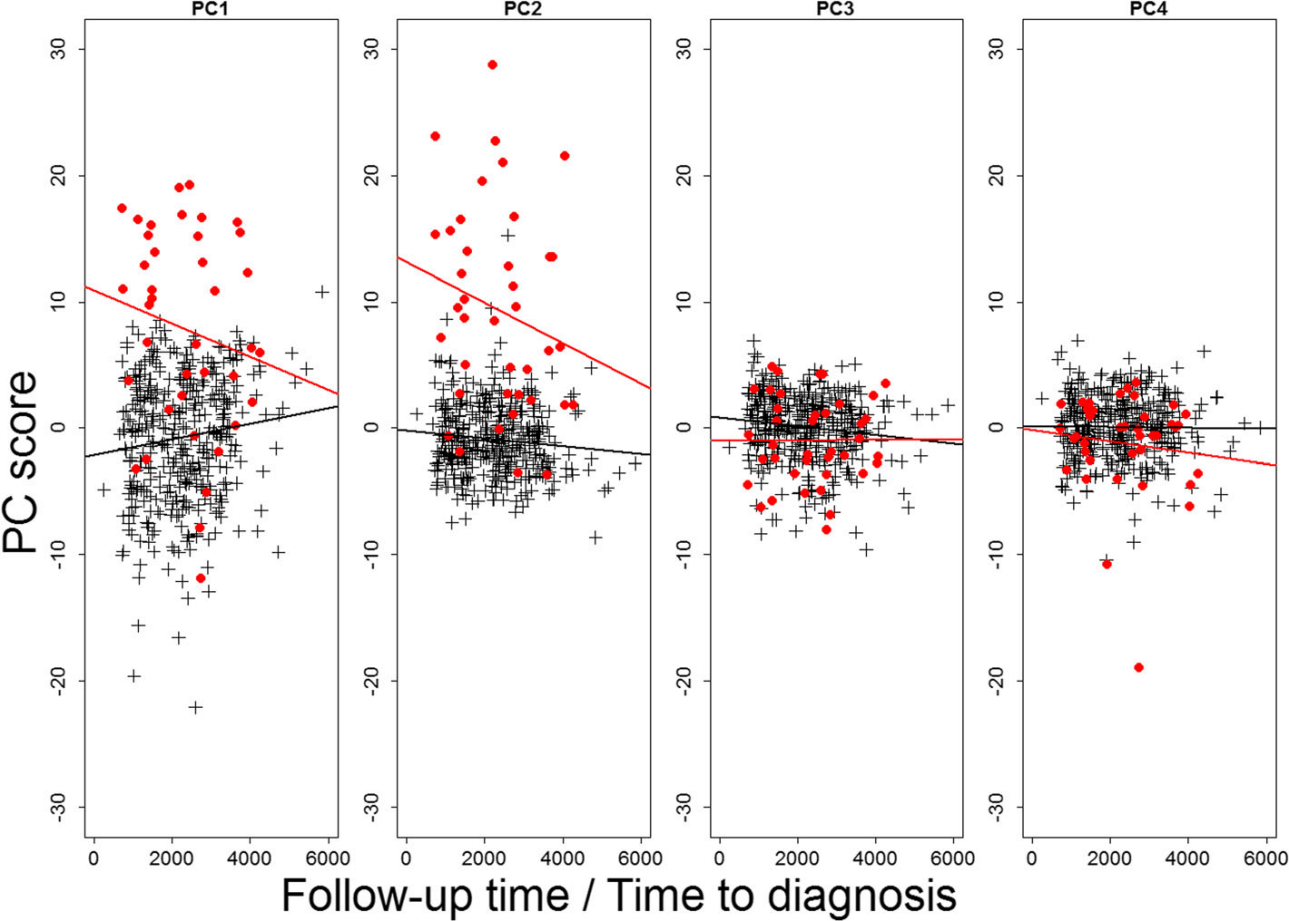
The association between follow-up time and principal components in similarly differentially expressed genes. Controls are shown in black, cases in red. Follow-up time in cases represents the time to diagnosis in days. Included genes are similarly differentially expressed in the prediagnostic study and the meta-analysis of clinical studies (groups ‘++’ and ‘--’). The first four principal components explained respectively 26.9, 16.5, 6.0 and 5.6%. When components were included in a linear model as the dependent variable, the time to diagnosis (in cases) showed a statistically significant association for PC1 (−1.55 × 10–3) and PC2 (−1.45 × 10–3) with respective *p* values of 0.023 and 0.007. Associations for PC3 and PC4 were not statistically significant

Both for markers that were overlapping in the prediagnostic study and in the meta-analysis of clinical studies (based on direction of effect and nominal significance) and for markers that reached only nominal significance in the prediagnostic study, the first two principal components were inversely associated with time to diagnosis, while principal components 3 and 4 were not significantly associated (Fig. 4 and Additional file 1: Figure S2). We observed high pairwise correlations between all four principal components derived in these gene sets (Additional file 1: Figure S3).

We studied 291 KEGG pathways for enrichment. KEGG pathway “hematopoietic cell lineage” was enriched within the genes that reached Bonferroni-significance in either the prediagnostic study or in the meta-analysis of the clinical studies (*p* value for enrichment: 5.07 × 10^−5^). Associations observed in the prediagnostic study and the meta-analysis for the 68 genes included in our study that are part of this pathway are listed in Additional file 1: Table S5. Eight genes were similarly differentially expressed (3 = ‘++’; 5 = ‘--’), 5 genes were exclusively seen prediagnostically (all ‘o+’). No genes were exclusively seen in the meta-analysis of clinical studies (either ‘+o’ or ‘-o’) or were dissimilarly differentially expressed genes (‘+−’ or ‘−+’). 55 genes were consistently non-significant between study types.

We assessed the overlap between our results and 44 putative CLL driver genes recently identified through whole-exome sequencing of 538 CLL and matched germline DNA samples [21] (Additional file 1: Table S6). Among the 41 genes that were also included in our analysis (genes SF3B1, CARD11, CHD2 were not), none achieved Bonferroni significance in either the prediagnostic or the meta-analysis of clinical studies. One gene (SAMHD1) was concurrently downregulated and achieved nominal significance in both the clinical and the prediagnostic study. Fourteen driver genes in the prediagnostic study and seven driver genes in the meta-analysis of clinical studies achieved nominal significance.

## Discussion

We assessed the concordance between gene-expression markers identified years before diagnosis with gene-expression markers of CLL identified at the clinical stage of the disease. By comparing these signals we aimed to elucidate the natural history of transcriptomic markers of CLL.

To our knowledge, this study was the first to combine transcriptomics data from clinical CLL studies in a meta-analytical framework. This method proved useful in identifying differentially expressed genes in clinical samples, with 116 differentially expressed genes observed. The variety in sampling tissue has probably increased the heterogeneity in signals which is likely to cause some attenuation. When only studies using peripheral blood samples were included in the meta-analyses the number of differentially expressed genes generally decreased, probably due to the lower number of subjects included.

The large overlap between markers identified in the meta-analysis of clinical studies and the prediagnostic study (60% of the genes identified in the meta-analysis) suggests that a differential gene expression pattern specific of disease can be detected in blood years before CLL diagnosis. This supports the assumption that differential expression observed before diagnosis is caused primarily by the presence of early disease at low concentration.

Further support for this assumption is provided by the inverse associations between principal components for levels of similarly differentially expressed genes (‘++’ and ‘--’) and time to diagnosis in cases. This association indicates that the underlying sources of variation become more apparent when approaching the time of diagnosis, which supports the hypothesis that this category includes genes for which differential expression is related to presence of diseased cells and/or etiological pathways leading to disease, and these are accumulating while approaching clinical onset. An association with time to diagnosis was also apparent for genes exclusively seen prediagnostically (‘o+’ and ‘o-’), which suggests that these genes were not functionally different from the similarly differentially expressed genes.

We hypothesize that the genes that were identified exclusively in the prediagnostic study operate in similar pathways as the genes that were identified in both the prediagnostic study and the meta-analysis of clinical studies. Support for this hypothesis was provided by the high pairwise correlations between principal components derived in these two sets of markers and by pathway enrichment analysis, in which genes exclusively identified in the prediagnostic study were not overrepresented compared to genes that were identified in both the prediagnostic study and the meta-analysis of clinical studies in the pathway that was enriched in our results (“hematopoietic cell lineage”).

Further support for the lack of functionally distinct groups of genes within the prediagnostic signals was provided by a post-hoc correlation analysis of gene expression in the prediagnostic study. The correlation analyses showed no apparent clusters between genes differentially expressed in the meta-analysis of clinical studies (groups ‘++’ and ‘--’) and genes that were not (groups ‘o+’ and ‘o-’) (Additional file 1: Figure S4). Under our hypothesis that gene groups ‘o+’ and ‘o-’ are not functionally different from gene groups ‘++’ and ‘--’, the lack of signal for the groups ‘o+’ and ‘o-’ gene groups in the meta-analysis of clinical studies is potentially explained by a lack of statistical power.

Detection of differential gene expression years before clinical diagnosis may be attributed to the presence of monoclonal B lymphocytosis (MBL). Although, in the absence of data describing the MBL status in each participant in our baseline data it was not possible to formally assess this assumption, in this light, it is interesting to contrast the prediagnostic signal we observed for CLL to the modest signal we observed for other types of B-cell lymphoma in the prediagnostic study, for which the contribution of a pre-lymphoma condition is less evident [1]. For multiple myeloma only two genes were found to be differentially expressed in future cases, whereas for diffuse large B cell lymphoma and follicular lymphoma no genes were differentially expressed. Multiple myeloma is preceded by premalignant monoclonal gammopathy of undetermined significance (MGUS). Where MBL has a very similar gene expression pattern to early-stage CLL [22] and progression depends on the number of CLL-type lymphocytes in the blood, [23] the progression of MGUS into MM is thought to require a more fundamental transformation of serum mIg levels and the bone-marrow plasma cell content induced by genetic alterations [24]. We are unaware of pre-lymphoma conditions preceding diffuse large B cell lymphoma and follicular lymphoma. However, the possibility that the overlapping signals for CLL were markers of susceptibility or markers of early biologic effect which remained present throughout the course of the disease cannot be excluded based on our analysis. Although we observed limited overlap between our results and 44 previously reported putative CLL driver genes, it is important to realize that this analysis pertained only to trans-acting relationships.

## Conclusions

We report concordance between gene expression signals observed in patients diagnosed with CLL and gene expression signals observed in future patients, years before they were diagnosed with CLL. This suggests that differentially expressed genes reflecting disease occurrence can be observed years before diagnosis and that these signals are retained throughout the disease course. No difference in association with time to diagnosis or functional annotation was observed between genes that were differentially expressed prediagnostically only and genes that were differentially expressed prediagnostically as well as clinically. This suggests that these signals may be involved in similar pathways, possible resulting from the presence of early disease. Although, there is no apparent clinical utility for early biomarkers of CLL, e.g. for screening purposes, the markers identified in this study could be used to follow the time-course of the disease, facilitating future deeper understanding of disease onset and factors that affect the disease. Furthermore our findings provide an opportunity to study CLL progression in more detail in future studies. Studies based on repeated sample collections before disease diagnosis should be performed to see if the identified markers here could be used for individual markers of disease progression.

## Additional file

Additional file 1: This file contains the supplementary tables and figures for this paper, including Tables S1-S6, and Figures S1-S4. (DOCX 1257 kb)

## Abbreviations

BMI: Body-mass index
CLL: Chronic lymphocytic leukemia
EBI: European Bioinformatics Institute
EPIC: European Prospective Investigation into Cancer and Nutrition
GEO: Gene Expression Omnibus
KEGG: Kyoto Encyclopedia of Genes and Genomes
MBL: Monoclonal B-cell lymphocytosis
NCBI: National Center for Biotechnology Information
MGUS: Monoclonal gammopathy of undetermined significance
NSHDS: The Northern Sweden Health and Disease Study
PBMC: Peripheral blood mononuclear cells
RNA: Ribonucleic acid
VIP: Västerbotten Intervention Programme
