# Supplementary material for: Exploring the nature of prediagnostic blood transcriptome markers of chronic lymphocytic leukemia by assessing their overlap with the transcriptome at the clinical stage
Source: BMC Genomics. 2017 Mar 20;18:239. doi: 10.1186/s12864-017-3627-4 (PMC5360061; doi:10.1186/s12864-017-3627-4)
Supplement: Additional file 1: — This file contains the supplementary tables and figures for this paper, including Tables S1-S6, and Figures S1-S4. (DOCX 1257 kb) [file 12864_2017_3627_MOESM1_ESM.docx]

**Table S1. Studies that explored genome-wide differential gene expression between cases and controls, as identified in GEO and ArrayExpress**

| **GEO accession** | **ArrayExpress accession** | **Reference** | **Platform** | **Disease** | **Samples (case/control)** | **Tissue (case/control)** |
| --- | --- | --- | --- | --- | --- | --- |
| GSE6691 | E-GEOD-6691 | (15) | Affymetrix HG-U133A Array | CLL | 11 / 5 | CLL samples / normal plasma cells from bone marrow |
| GSE8835 | E-GEOD-8835 | (16) | Affymetrix HG-U133A Array | CLL | 22 / 12 | CD4+ T cells from peripheral blood ^A^ |
| GSE13159 | E-GEOD-13159 | (17) | Affymetrix HG-U133 Plus 2.0 Array | CLL | 448 / 74 ^B^ | Mononuclear cells from peripheral blood / healthy bone marrow specimens |
| GSE18026 | E-GEOD-18026 | (18) | Affymetrix HG-U133A Array | CLL | 5 / 2 | Peripheral blood mononuclear CLL cells / B cells from peripheral blood |
| GSE19147 | E-GEOD-19147 | (19) | Affymetrix HG-U133A Array | CLL | 25 / 8 | CD3+ T cells from peripheral blood ^A^ |
| GSE26725 | E-GEOD-26725 | (20) | Affymetrix HG-U133 Plus 2.0 Array | CLL | 12 / 5 | B-CLL / CD19+ B cells from peripheral blood |
| GSE28107 | E-GEOD-28107 | N/A | Affymetrix HG-U133A Array | CLL | 3 / 3 | T cells from peripheral blood ^A^ |
| GSE32018 | E-GEOD-32018 | (21) | Agilent Whole Human Genome Microarray 4x44K G4112F | CLL | 17 / 7 | Freshly frozen lymph nodes ^A^ |
| GSE35179 | E-GEOD-35179 | (22) | Affymetrix HG-U95A Array | CLL | 5 / 5 | Monocytes from peripheral blood ^A^ |

^A^ Similar tissues were used for cases and controls ^B^ Controls were ‘participants with healthy bone marrow specimens and nonleukemia conditions, such as megaloblastic anemia, hemolysis, iron deficiency, or idiopathic thrombocytopenic purpura’.

**Table S2. The 25 genes with lowest *P* values^a^ from the meta-analyses of clinical studies of Chronic Lymphocytic Leukemia.**

| **Rank** | **Gene** | **Z-score** | ***P* value** |  |  |  |
| --- | --- | --- | --- | --- | --- | --- |
| 1 | *TCF7L2* | -11.92 | 9.30E-33 |  |  |  |
| 2 | *MAN1C1 ^b^* | -9.99 | 1.74E-23 |  |  |  |
| 3 | *EMP1 ^A^* | -9.2 | 3.49E-20 |  |  |  |
| 4 | *ITM2B* | -8.68 | 3.95E-18 |  |  |  |
| 5 | *SMAD7 ^c^* | -8.36 | 1.11E-16 |  |  |  |
| 6 | *FHIT ^b^* | -8.12 | 4.44E-16 |  |  |  |
| 7 | *FAM49B ^b^* | -8.09 | 6.66E-16 |  |  |  |
| 8 | *FHL1* | -8.02 | 1.11E-15 |  |  |  |
| 9 | *ASAP1-IT1 ^d^* | -7.99 | 1.33E-15 |  |  |  |
| 10 | *LYZ ^b^* | -7.87 | 3.55E-15 |  |  |  |
| 11 | *IFIT1* | -7.69 | 1.43E-14 |  |  |  |
| 12 | *KLF7* | -7.61 | 2.73E-14 |  |  |  |
| 13 | *CPVL ^b^* | -6.97 | 3.12E-12 |  |  |  |
| 14 | *LSM2 ^b^* | 6.89 | 5.57E-12 |  |  |  |
| 15 | *PILRA* | -6.84 | 8.17E-12 |  |  |  |
| 16 | *FBXL15* | 6.81 | 9.65E-12 |  |  |  |
| 17 | *EFHC2 ^c^* | -6.73 | 1.75E-11 |  |  |  |
| 18 | *SPG20 ^b^* | -6.67 | 2.49E-11 |  |  |  |
| 19 | *CCL20 ^d^* | -6.56 | 5.28E-11 |  |  |  |
| 20 | *SPATA6 ^b^* | -6.56 | 5.56E-11 |  |  |  |
| 21 | *SCO2 ^b^* | -6.49 | 8.33E-11 |  |  |  |
| 22 | *SEC63* | -6.41 | 1.49E-10 |  |  |  |
| 23 | *AIF1 ^b^* | -6.36 | 2.07E-10 |  |  |  |
| 24 | *NOTCH3* | -6.28 | 3.39E-10 |  |  |  |
| 25 | *PGAP1 ^b^* | -6.19 | 6.20E-10 |  |  |  |

^a^ Bonferroni-corrected thresholds of statistical significance was 4.20E-6.
^b^ Gene was significant in prediagnostic study with *P* value < 0.05.
^c^ Gene was significant in prediagnostic study after Bonferroni correction.
^d^ Gene was not studied in prediagnostic study.

**Table S3. Top 25 genes most significantly associated genes^a^ with Chronic Lymphocytic Leukemia. Results from replication on a gene level of the prediagnostic study of Chadeau-Hyam et al. (collapsed by gene).**

|  | | | |
| --- | --- | --- | --- |
| **Rank** | **Gene** | **f ^b^** | ***P* value^a^** |
| 1 | *ABCA6* | 4.06 | 1.19E-90 |
| 2 | *ARHGAP44* | 4.58 | 4.33E-69 |
| 3 | *WNT3* | 3.87 | 1.34E-53 |
| 4 | *ZBTB32* | 2.4 | 3.36E-49 |
| 5 | *CDK14* | 1.82 | 1.37E-47 |
| 6 | *CNR2* | 1.34 | 2.67E-47 |
| 7 | *TCF4* | 1.88 | 1.11E-45 |
| 8 | *KCNN4* | 1.69 | 6.21E-45 |
| 9 | *FCRL2* | 1.84 | 2.40E-40 |
| 10 | *RHOBTB2* | 1.28 | 5.17E-39 |
| 11 | *MIR155HG* | 1.85 | 6.92E-39 |
| 12 | *FCRL5* | 2.24 | 4.46E-38 |
| 13 | *METTL8* | 1.35 | 1.02E-37 |
| 14 | *CD24* | 1.59 | 5.57E-37 |
| 15 | *SOBP* | 2.12 | 5.32E-36 |
| 16 | *FCER2* | 1.74 | 4.70E-34 |
| 17 | *CELSR1* | 1.75 | 5.58E-34 |
| 18 | *COL9A2* | 1.2 | 3.32E-31 |
| 19 | *SYT17* | 1.41 | 1.05E-30 |
| 20 | *KLHL14* | 1.94 | 2.13E-30 |
| 21 | *BLK* | 1.63 | 3.52E-30 |
| 22 | *CXXC5* | 0.95 | 1.76E-29 |
| 23 | *RASGRP3* | 1.48 | 1.12E-28 |
| 24 | *ZCCHC18* | 1.14 | 4.95E-28 |
| 25 | *FCRLA* | 1.7 | 7.80E-28 |

^a^ Bonferroni-corrected threshold of statistical significance was 3.20E-6
^b^ Fold-change (f) is derived from the regression coefficient (β) from the linear mixed model: f = 2^β^

| Upregulated genes achieving genome-wide significance in both studies (n=1) | \| COCH \| \| --- \| |
| --- | --- | --- |
| Upregulated genes  achieving  genome-wide  significance only in  the prospective  study (n=227) | \| ABCA6 \| C11orf24 \| COL9A3 \| GATM \| KNTC1 \| PLCG2 \| SMARCB1 \| TP53I3 \| \| --- \| --- \| --- \| --- \| --- \| --- \| --- \| --- \| \| ABCC8 \| C1orf56 \| CPEB1 \| GCLC \| LAX1 \| PLEKHF2 \| SMARCC2 \| TRAM2 \| \| ACADM \| C20orf195 \| CSH2 \| GGA2 \| LHPP \| PMAIP1 \| SMC6 \| TRAPPC2L \| \| ACP5 \| C5orf45 \| CTLA4 \| GH1 \| LRMP \| PNOC \| SOBP \| TTC28 \| \| ACSF2 \| C9orf91 \| CTSH \| HEATR6 \| LRRK1 \| POU2AF1 \| SP140 \| TTN \| \| ADAM28 \| CAPN3 \| CXCR5 \| HIBCH \| MAGEF1 \| PRKCB \| SPATS2L \| UGCG \| \| ADAM29 \| CBFA2T3 \| CYB5R2 \| HIVEP1 \| MARCH1 \| PRKCE \| SPIB \| UGGT2 \| \| ADAMDEC1 \| CCDC88A \| DCLK2 \| HLA-DMA \| MARS \| PTPN2 \| ST14 \| UGT8 \| \| AFF3 \| CD19 \| DENND5B \| HLA-DMB \| MBD4 \| QRSL1 \| ST6GAL1 \| UPF3A \| \| AFTPH \| CD200 \| DEPDC5 \| HLA-DOA \| MCM5 \| RALGPS1 \| STAMBPL1 \| USP11 \| \| AGPAT5 \| CD24 \| DGKG \| HLA-DOB \| MEF2C \| RALGPS2 \| STAP1 \| USP6NL \| \| AIM2 \| CD37 \| DLGAP4 \| HRK \| METTL7A \| RASGRP3 \| SWAP70 \| VAV2 \| \| APBB2 \| CD40 \| DMXL1 \| HS3ST1 \| METTL8 \| RHOBTB2 \| SYK \| VOPP1 \| \| APOM \| CD70 \| DND1 \| ID3 \| MICAL3 \| RIC3 \| SYPL1 \| VPREB3 \| \| APPL1 \| CD72 \| DNMBP \| IFT57 \| MMP11 \| RNFT2 \| SYT17 \| WDR11 \| \| ARHGAP24 \| CD74 \| E2F5 \| IL24 \| MTSS1 \| ROR1 \| TBC1D1 \| WDR91 \| \| ARHGAP44 \| CD79B \| ENPP2 \| IL2RA \| MYL6B \| RRAS2 \| TBC1D22A \| WNT3 \| \| ARID5B \| CD82 \| EPS8L2 \| IL4R \| MYO1E \| SCRN1 \| TCF3 \| ZBTB24 \| \| ARMCX2 \| CDC16 \| FAIM3 \| IL7 \| NCOA3 \| SEL1L3 \| TCF4 \| ZBTB32 \| \| B4GALT1 \| CDK14 \| FAM3C \| INPP5F \| NEK3 \| SETBP1 \| TCL1A \| ZNF107 \| \| BACE2 \| CELSR1 \| FARP2 \| IRF8 \| NFATC1 \| SFMBT1 \| TCTN1 \| ZNF266 \| \| BACH2 \| CELSR3 \| FCER2 \| ISCU \| NOC3L \| SH3BP5 \| TFEB \| ZNF415 \| \| BAIAP3 \| CENPJ \| FCGR2B \| IZUMO4 \| NUP88 \| SHMT2 \| TFRC \| ZNF506 \| \| BCAR3 \| CHD7 \| FCRL2 \| KCNH2 \| OSBPL10 \| SIPA1L3 \| THNSL2 \| ZNF821 \| \| BCAS4 \| CLASRP \| FGGY \| KCNN4 \| PARP3 \| SLC23A2 \| THOP1 \|  \| \| BCL11A \| CNR2 \| FIG4 \| KLF8 \| PAWR \| SLC2A11 \| TIMELESS \|  \| \| BICD1 \| CNTNAP2 \| FLT1 \| KLHL29 \| PCDH9 \| SLC35E3 \| TMEM19 \|  \| \| BLK \| COL4A4 \| GAB1 \| KLK1 \| PEBP1 \| SLC35F2 \| TNFRSF13B \| \| \| BLNK \| COL9A2 \| GATAD1 \| KMO \| PKIG \| SLC9A7 \| TNS4 \|  \| |
| Upregulated genes  achieving genome-  wide significance  only in the clinical  study (n=18) | \| C3orf14 \| \| DNAJC17 \| \| FBXO4 \| LSM2 \| NETO2 \| \| \| SPAG7 \| WEE1 \| ZNF277 \| \| --- \| --- \| --- \| --- \| --- \| --- \| --- \| --- \| --- \| --- \| --- \| --- \| \| CIITA \| \| FBXL15 \| \| KIAA0226 \| MCTP2 \| RASSF7 \| \| \| UTS2 \| YPEL1 \| ZNF443 \| \| DCAF17 \|  \| \|  \| \|  \| \|  \|  \| \|  \|  \| |

**Table S4. Concurrently up- or downregulated genes that reached Bonferroni-significance in either the prediagnostic study or in the meta-analysis of clinical studies.**

| Downregulated genes achieving genome-wide significance in both studies (n=8) | \| ARHGAP32 \| EFHC2 \| FAM134B \| KLF3 \| MAFB \| RAB33A \| SCML1 \| SMAD7 \| \| --- \| --- \| --- \| --- \| --- \| --- \| --- \| --- \| |
| --- | --- | --- | --- | --- | --- | --- | --- | --- | --- |
| Downregulated genes achieving genome-wide significance only in the prospective study (n=105) | \| ADA \| CDR2 \| FAM169A \| ICOS \| METRN \| PDGFA \| S1PR2 \| TNFRSF4 \| \| --- \| --- \| --- \| --- \| --- \| --- \| --- \| --- \| \| AFAP1 \| CLCF1 \| FAM50B \| IL15 \| MFAP3L \| PELI2 \| SEMA4C \| TNIK \| \| AKAP7 \| CLIP2 \| FYB \| IL3RA \| MITF \| PKP4 \| SH2D2A \| TRIB2 \| \| AOAH \| CORO1C \| FYN \| IQGAP2 \| MLLT3 \| PLCB1 \| SMPD3 \| TSPAN13 \| \| AQP3 \| CORO2A \| GCH1 \| ITGA6 \| MMRN1 \| PLCD1 \| SOCS2 \| ULBP2 \| \| ARHGEF5 \| CTSW \| GLA \| ITGAL \| MPRIP \| PPFIBP1 \| SPN \| VANGL1 \| \| ASAP2 \| CXorf57 \| GLDC \| ITK \| MRAS \| PPM1A \| SPON1 \| XPNPEP1 \| \| ASGR1 \| CYB561 \| GNAQ \| KCTD15 \| MREG \| PRKCA \| SPSB1 \|  \| \| BCL11B \| DAB2 \| GPD1L \| KIAA0513 \| MYC \| PRKCQ \| SSX2IP \|  \| \| BUB1 \| DNM1 \| GPR20 \| LARGE \| MYCT1 \| PTP4A3 \| STOM \|  \| \| CD244 \| DNM3 \| GPRC5B \| LEPR \| NAP1L2 \| PTPRK \| SVIL \|  \| \| CD36 \| EPB41L3 \| HIST1H2AC \| LMNA \| NAT8B \| RARRES3 \| SYNJ1 \|  \| \| CD3E \| EPPK1 \| HOPX \| LOXL1 \| NECAB3 \| RGL1 \| TBC1D4 \|  \| \| CDH1 \| FAM110B \| HS3ST3B1 \| MAF \| NECAP1 \| RORA \| TMEM45A \| \| |
| Downregulated genes achieving genome-wide significance only in the clinical study (n=66) | \| A2M \| CST3 \| FBP1 \| HDC \| KLRB1 \| MEIS1 \| PTGER3 \| TCF7L2 \| \| --- \| --- \| --- \| --- \| --- \| --- \| --- \| --- \| \| ABCC3 \| DDO \| FHIT \| HIF1A \| LAMB2 \| NOTCH3 \| RPS6KA4 \| TLR5 \| \| AHR \| DEGS1 \| FHL1 \| IFI44 \| LAMP3 \| OGFRL1 \| RQCD1 \| TREM1 \| \| AIF1 \| DLK1 \| FLVCR2 \| IFI44L \| LPHN1 \| PCSK5 \| RTN2 \|  \| \| ANKRD55 \| DNASE1L3 \| FOXN2 \| IFI6 \| LPIN1 \| PDE5A \| SCARB2 \|  \| \| C1orf54 \| ELK3 \| GDPD5 \| IFIT1 \| LRRC6 \| PGAP1 \| SCO2 \|  \| \| CAMK4 \| EMP1 \| GNG11 \| IGF1R \| LYZ \| PILRA \| SLCO3A1 \|  \| \| CD7 \| EPHB2 \| GPR125 \| ITM2B \| MAN1C1 \| PLCL1 \| SPATA6 \|  \| \| CPVL \| FAM49B \| GSTM5 \| KLF7 \| MAP7D1 \| PLXDC1 \| SPG20 \|  \| |

**Table S5. Estimates^a^ and associated *p* values^b^ for 68 genes involved in KEGG pathway ‘hematopoietic cell lineage’.**

| **Gene**  **symbol** | **Estimate meta-analysis of clinical studies** | **Estimate pre-diagnostic study** |
| --- | --- | --- |
| ANPEP | -1.8636  (p=6.24E-02) | -0.2281  (p=1.03E-01) |
| CD14 | -1.2463  (p=2.13E-01) | -0.3086  (p=3.65E-03) |
| CD19 | 1.5491  (p=1.21E-01) | **1.4827  (p=5.41E-27)** |
| CD1A | -2.9502  (p=3.18E-03) | -0.4757  (p=3.22E-05) |
| CD1B | -1.8904  (p=5.87E-02) | 0.1287  (p=2.58E-01) |
| CD1C | -2.7146  (p=6.64E-03) | -0.4329  (p=3.14E-05) |
| CD1D | -1.7328  (p=8.31E-02) | -0.1472  (p=1.96E-01) |
| CD2 | -0.4594  (p=6.46E-01) | -0.3676  (p=1.35E-03) |
| CD22 | 0.0477  (p=9.62E-01) | 0.5392  (p=7.63E-06) |
| CD24 | 1.5862  (p=1.13E-01) | **1.5877  (p=5.57E-37)** |
| CD33 | -2.9683  (p=2.99E-03) | -0.3139  (p=5.00E-03) |
| CD36 | -2.2730  (p=2.30E-02) | **-0.4318  (p=6.78E-07)** |
| CD37 | 0.9143  (p=3.61E-01) | **0.5052  (p=1.05E-07)** |
| CD38 | -2.7191  (p=6.55E-03) | -0.5107  (p=7.59E-06) |
| CD3D | -1.2975  (p=1.94E-01) | -0.3929  (p=4.41E-04) |
| CD3E | -2.6586  (p=7.85E-03) | **-0.4240  (p=1.30E-07)** |
| CD3G | -0.0939  (p=9.25E-01) | -0.3893  (p=1.43E-03) |
| CD4 | -3.2737  (p=1.06E-03) | -0.1854  (p=2.15E-01) |
| CD44 | -2.4466  (p=1.44E-02) | -0.1530  (p=3.51E-02) |
| CD5 | 1.3417  (p=1.80E-01) | 0.0273  (p=6.73E-01) |
| CD55 | -1.9364  (p=5.28E-02) | -0.0421  (p=6.91E-01) |
| CD59 | -1.1412  (p=2.54E-01) | -0.0859  (p=1.93E-01) |
| CD7 | **-5.0946  (p=3.50E-07)** | **-0.3603  (p=1.38E-05)** |
| CD8A | 0.1547  (p=8.77E-01) | -0.4899  (p=5.79E-04) |
| CD9 | -1.4907  (p=1.36E-01) | -0.5662  (p=2.31E-04) |
| CR1 | -2.7188  (p=6.55E-03) | -0.1888  (p=9.05E-02) |
| CR2 | -0.9734  (p=3.30E-01) | -0.0285  (p=7.97E-01) |
| CSF1 | -1.3936  (p=1.63E-01) | -0.2117  (p=6.15E-02) |
| CSF1R | -2.8510  (p=4.36E-03) | -0.3779  (p=1.64E-03) |
| CSF2RA | -1.7763  (p=7.57E-02) | -0.2463  (p=2.55E-02) |
| CSF3R | -2.0466  (p=4.07E-02) | -0.4416  (p=4.50E-03) |
| EPOR | -1.6857  (p=9.19E-02) | -0.1791  (p=4.06E-03) |
| FCER2 | 2.2965  (p=2.16E-02) | **1.7400  (p=4.70E-34)** |
| FLT3 | -1.3779  (p=1.68E-01) | 0.2854  (p=2.91E-02) |
| FLT3LG | -1.5889  (p=1.12E-01) | -0.4250  (p=2.00E-05) |
| GP1BA | -2.7511  (p=5.94E-03) | -0.4092  ( p=9.25E-05) |
| GP9 | -2.5080  (p=1.21E-02) | -0.1958  (p=5.61E-03) |
| GYPA | -0.8749  (p=3.82E-01) | -0.1984  (se=0.1300; p=1.22E-01) |
| HLA-DRA | 1.2619  (p=2.07E-01) | 0.2869  (p=1.85E-02) |
| HLA-DRB4 | 1.3785  (p=1.68E-01) | 0.1486  (p=2.77E-01) |
| IL11RA | 0.5592  (p=5.76E-01) | -0.1572  (p=3.82E-02) |
| IL1B | -2.7230  (p=6.47E-03) | -0.6221  (p=1.89E-02) |
| IL1R1 | -2.6733  (p=7.51E-03) | -0.2181  (p=1.05E-02) |
| IL1R2 | -1.1781  (p=2.39E-01) | -0.2261  (p=1.38E-01) |
| IL2RA | 2.2693  (p=2.32E-02) | **0.5098  (p=2.22E-06)** |
| IL3RA | -3.2087  (p=1.33E-03) | **-0.4720  (p=7.15E-07)** |
| IL4 | -2.2245  (p=2.61E-02) | -0.4141  (p=5.37E-02) |
| IL4R | 1.6152  (p=1.06E-01) | **0.8911  (p=4.08E-13)** |
| IL5RA | -1.7380  (p=8.22E-02) | -0.2482  (p=7.68E-02) |
| IL6 | -2.3769  (p=1.75E-02) | -0.6880  (p=6.71E-05) |
| IL6R | -3.2445  (p=1.18E-03) | -0.1846  (p=8.85E-02) |
| IL7 | 2.7378  (p=6.18E-03) | **1.2341  (p=2.37E-23)** |
| IL7R | -1.3704  (p=1.71E-01) | -0.4830  (p=1.73E-04) |
| IL9R | -0.3613  (p=7.18E-01) | -0.0589  (p=6.27E-01) |
| ITGA1 | -0.8777  (p=3.80E-01) | -0.4908  (p=4.02E-03) |
| ITGA2 | -3.9527  (p=7.73E-05) | -0.5135  (p=4.69E-03) |
| ITGA2B | -1.8168  (p=6.93E-02) | -0.3758  (p=5.95E-03) |
| ITGA4 | -2.4605  (p=1.39E-02) | -0.4137  (p=9.60E-06) |
| ITGA5 | -3.0548  (p=2.25E-03) | -0.2570  (p=6.37E-03) |
| ITGA6 | -2.3596  (p=1.83E-02) | **-0.5606  (p=2.98E-12)** |
| ITGAM | -1.3744  (p=1.69E-01) | -0.2243  (p=5.64E-03) |
| ITGB3 | -4.3626  (p=1.29E-05) | -0.5040  (p=2.94E-04) |
| KIT | -1.9026  (p=5.71E-02) | -0.3918  (p=3.92E-04) |
| MME | -1.6173  (p=1.06E-01) | -0.2692  (p=2.50E-01) |
| MS4A1 | 1.2524  (p=2.10E-01) | 0.5296  (p=4.96E-05) |
| TFRC | 0.3207  (p=7.48E-01) | **0.4343  (p=6.32E-08)** |
| THPO | -0.8664  (p=3.86E-01) | -0.0211  (p=8.83E-01) |
| TNF | 0.4880  (p=6.26E-01) | -0.3290  (p=4.86E-02) |

^a^ Highlighted green is downregulated, Highlighted red is upregulated among cases.

^b^ Bold estimates indicate genome wide significance.

**Table S6. Estimates^a^ and associated *p* values^b^ for 44 putative driver genes of Chronic Lymphocytic Leukemia^c^.**

| **Gene symbol** | **Estimate meta-analysis of clinical studies** | **Estimate pre-diagnostic study** |
| --- | --- | --- |
| ASXL1 | 0.5913  (p=5.54E-01) | 0.1949  (p=3.52E-03) |
| ATM | -0.7035  (p=4.82E-01) | -0.0128  (p=8.94E-01) |
| BAZ2A | 0.8895  (p=3.74E-01) | 0.0199  (p=8.44E-01) |
| BCOR | 2.4297  (p=1.51E-02) | -0.0337  (p=7.04E-01) |
| BIRC3 | 0.8783  (p=3.80E-01) | 0.3242  (p=7.43E-03) |
| BRAF | 0.3594  (p=7.19E-01) | 0.1235  (p=3.72E-02) |
| BRCC3 | 2.1311  (p=3.31E-02) | 0.0893  (p=1.22E-01) |
| CHEK2 | 0.2397  (p=8.11E-01) | 0.1517  (p=1.62E-01) |
| DDX3X | -1.0340  (p=3.01E-01) | -0.2288  (p=3.08E-03) |
| DYRK1A | -1.8317  (p=6.70E-02) | -0.0105  (p=8.92E-01) |
| EGR2 | 1.1101  (p=2.67E-01) | -0.0316  (p=8.91E-01) |
| ELF4 | -0.6161  (p=5.38E-01) | -0.0756  (p=3.65E-01) |
| EWSR1 | 2.3042  (p=2.12E-02) | 0.0123  (p=8.67E-01) |
| FAM50A | 2.0134  (p=4.41E-02) | 0.0232  (p=7.10E-01) |
| FBXW7 | 0.3845  (p=7.01E-01) | -0.0946  (p=2.41E-01) |
| FUBP1 | 1.0681  (p=2.85E-01) | -0.0167  (p=9.05E-01) |
| GNB1 | 1.5337  (p=1.25E-01) | -0.1167  (p=3.16E-02) |
| HIST1H1B | -1.0680  (p=2.86E-01) | 0.3158  (p=3.76E-02) |
| HIST1H1E | 1.6297  (p=1.03E-01) | 0.4630  (p=7.17E-04) |
| IKZF3 | 1.6659  (p=9.57E-02) | 0.5728  (p=7.35E-06) |
| IRF4 | 0.2948  (p=7.68E-01) | 0.3782  (p=4.48E-06) |
| KRAS | -0.4928  (p=6.22E-01) | -0.1401  (p=2.12E-01) |
| MAP2K1 | -0.2087  (p=8.35E-01) | -0.0681  (p=4.50E-01) |
| MED12 | 1.0305  (p=3.03E-01) | -0.1422  (p=1.94E-01) |
| MGA | 2.6213  (p=8.76E-03) | 0.0356  (p=4.78E-01) |
| MYD88 | -0.2085  (p=8.35E-01) | -0.1518  (p=3.47E-02) |
| NOTCH1 | 0.6043  (p=5.46E-01) | -0.1803  (p=2.01E-01) |
| NRAS | 0.2847  (p=7.76E-01) | -0.1938  (p=2.09E-02) |
| NXF1 | -0.5209  (p=6.02E-01) | -0.0245  (p=7.52E-01) |
| PIM1 | 1.5087  (p=1.31E-01) | -0.2464  (p=4.33E-03) |
| POT1 | 2.4507  (p=1.43E-02) | 0.1126  (p=2.34E-01) |
| PTPN11 | 0.8652  (p=3.87E-01) | 0.0159  (p=7.65E-01) |
| RPS15 | -0.4819  (p=6.30E-01) | 0.0522  (p=3.11E-01) |
| SAMHD1 | -2.9200  (p=3.50E-03) | -0.2839  (p=5.30E-03) |
| SF3B1 | 1.0287  (p=3.04E-01) | 0.0609  (p=4.51E-01) |
| TP53 | 0.0776  (p=9.38E-01) | -0.0883  (p=5.73E-01) |
| TRAF2 | 1.6398  (p=1.01E-01) | 0.0668  (p=5.85E-01) |
| TRAF3 | 0.6928  (p=4.88E-01) | 0.3741  (p=5.63E-05) |
| XPO1 | 0.6932  (p=4.88E-01) | 0.0081  (p=9.25E-01) |
| XPO4 | 0.0237  (p=9.81E-01) | 0.0082  (p=9.21E-01) |
| ZMYM3 | 0.8635  (p=3.88E-01) | 0.0412  (p=5.01E-01) |

^a^ Highlighted green is downregulated, Highlighted red is upregulated.

^b^ Bold estimates indicate genome wide significance.

^c^ As reported by Landau, D. A. et al. Mutations driving CLL and their evolution in progression and relapse. Nature 526, 525–30 (2015).

**Figure S1. Number of studies found on GEO and ArrayExpress, assessed for eligibility, and included in the review, with reasons for exclusions at each stage.**

**
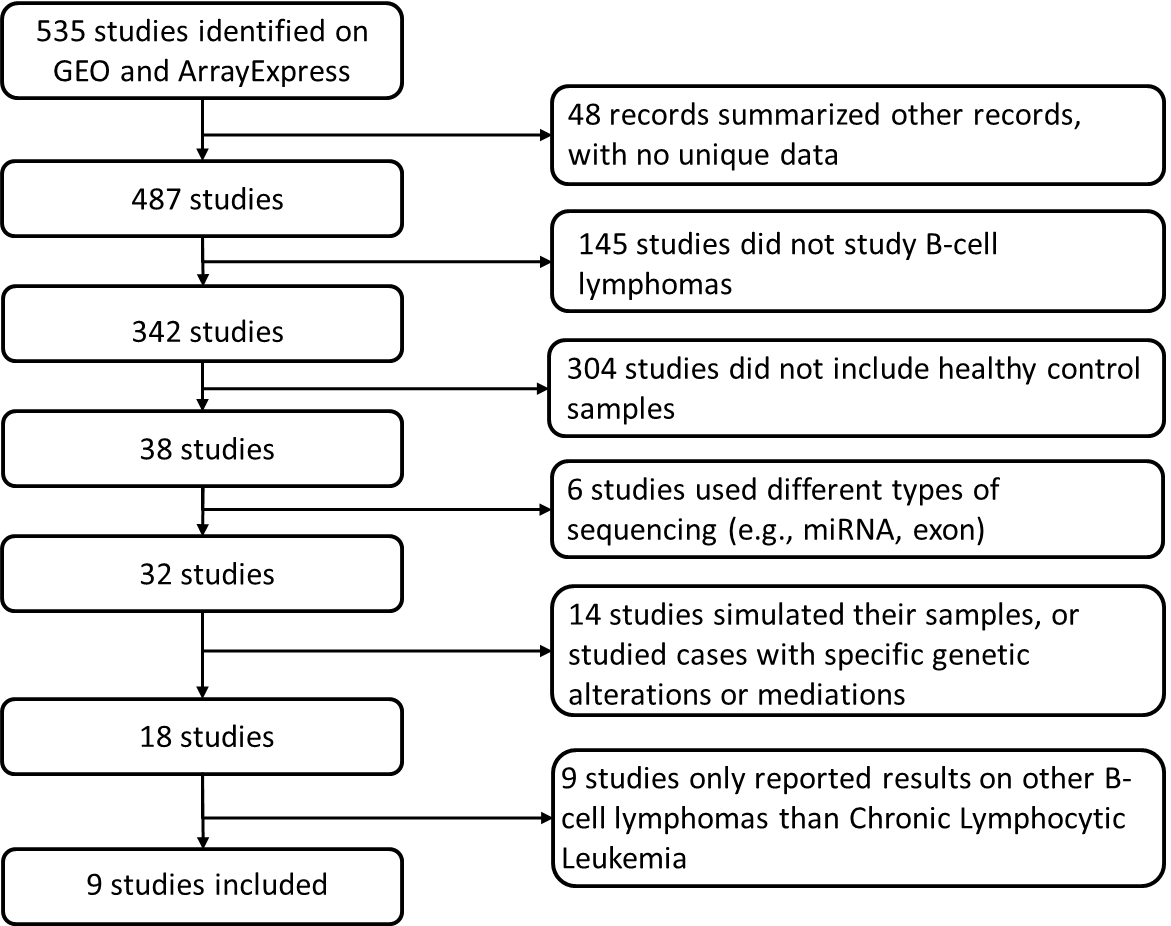
**

**Figure S2. Scatterplots showing the association between the follow-up time (or time to diagnosis for cases, shown in red) and the principal components in the genes exclusively seen in the prediagnostic study (‘o+’ and ‘o-’).**


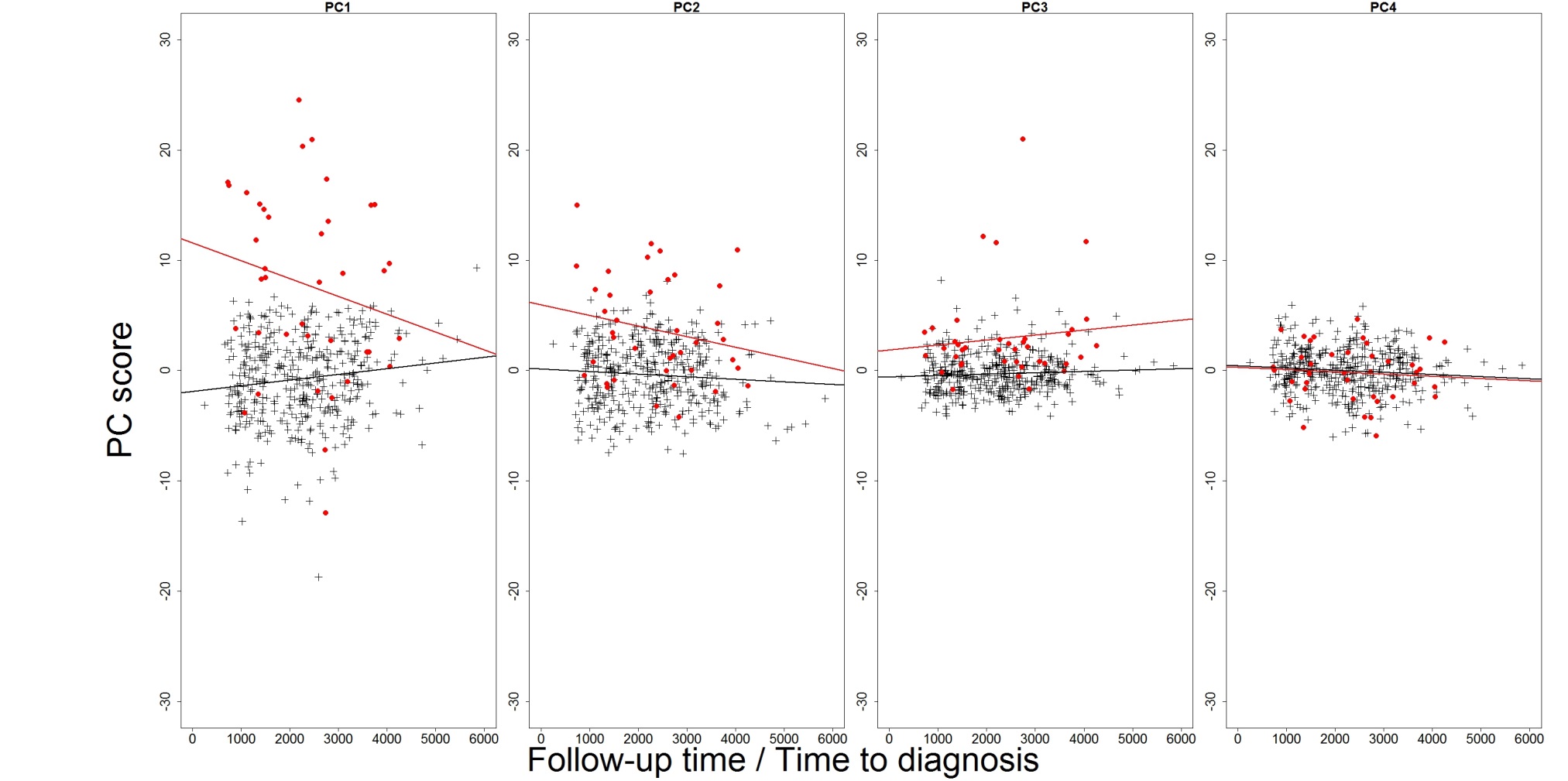


The first four principal components explained respectively 29.3%, 13.0%, 6.1% and 5.1%. When components were included in a linear model as the dependent variable, the time to diagnosis (in cases) showed a statistically significant association for PC1 (-1.74 x 10^-3^) and PC2 (-8.26 x 10^-4^) with respective *P* values of 0.007 and 0.038. Associations for PC3 and PC4 were not statistically significant.

**Figure S3. Correlation plots of principal components (PC) explaining more than 5% of the variance within similarly differentially expressed genes (‘++’ and ‘--’) and genes exclusively seen prediagnostically (‘o+’ and ‘o-’).**


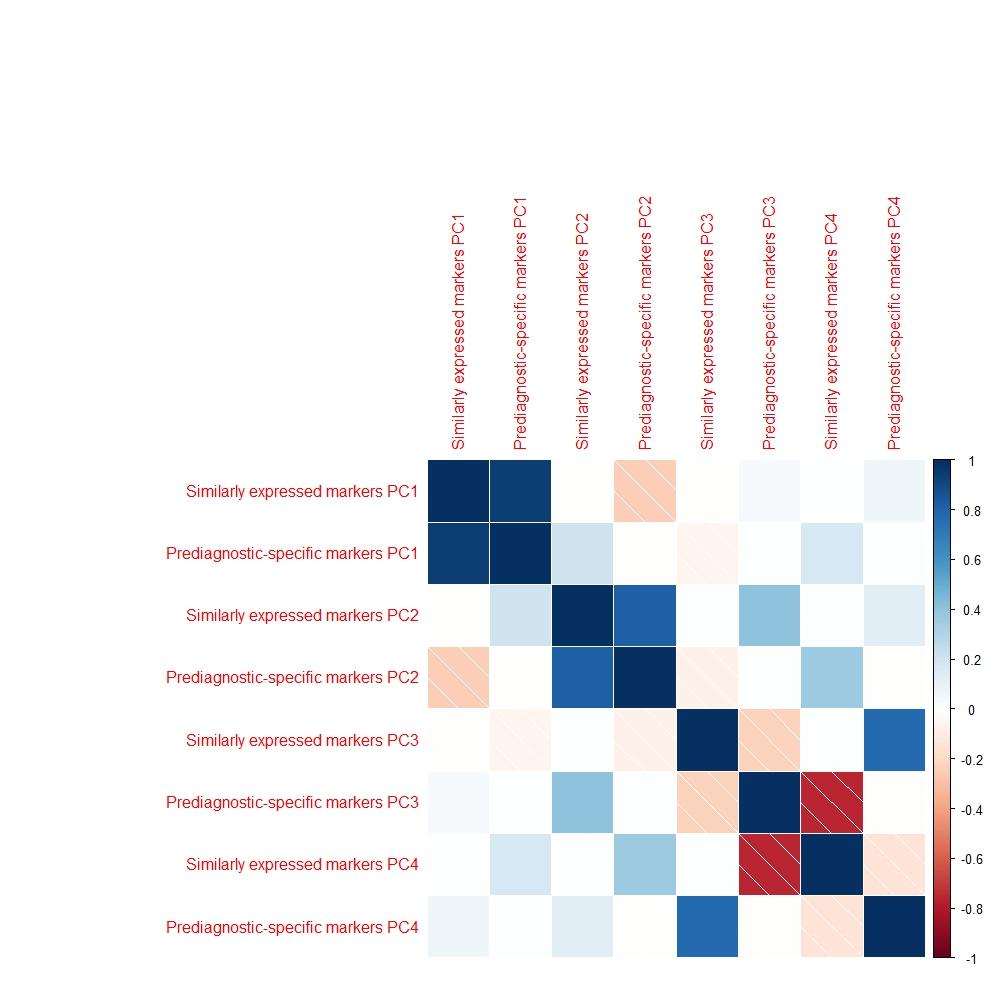


**Figure S4. Correlation plots of differentially expressed prediagnostic markers, comparing genes that were similarly differentially expressed in the meta-analysis of clinical studies (‘++’, ‘--’) with genes that were not differentially expressed in the meta-analysis of clinical studies (‘o+’, ‘o-’).**

**
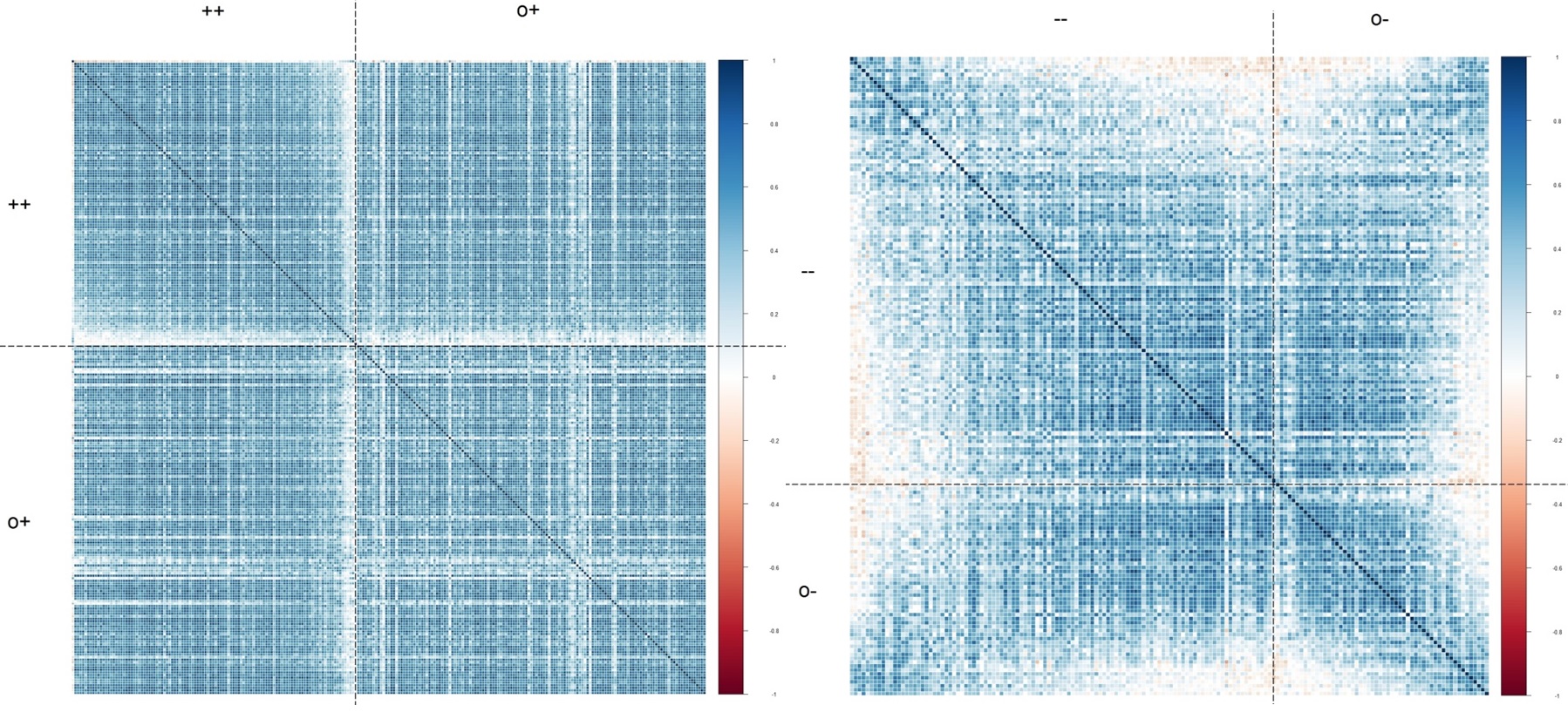
**

Scores depicted are Pearson’s correlation coefficients ranging from -1 (deep red) to 1 (deep blue)
